# Supplementary material for: Testicular “Inherited Metabolic Memory” of Ancestral High-Fat Diet Is Associated with Sperm sncRNA Content
Source: Biomedicines. 2022 Apr 15;10(4):909. doi: 10.3390/biomedicines10040909 (PMC9027117; doi:10.3390/biomedicines10040909)
Supplement: Supplementary file 1 [file biomedicines-10-00909-s001.zip › biomedicines-1651197-supplementary.pdf]

Supporting information

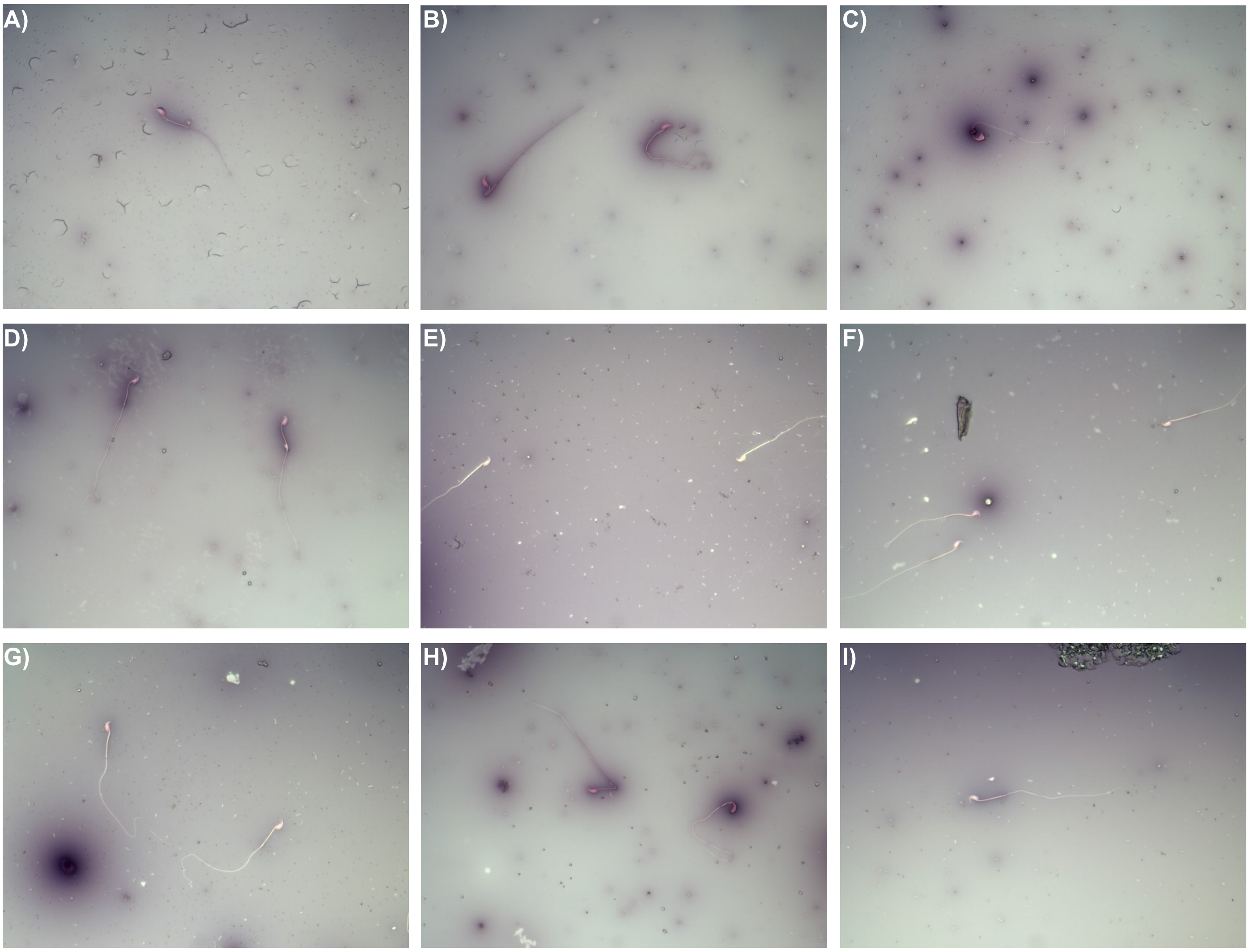

Figure S1: Mice epididymal sperm swabs stained by eosin-nigrosin dye and observed by x400 magnification using an optical microscope. Epididymal spermatozoa were obtained immediately after the collection of the epididymis by swim-out. Shortly, the epididymis was collected in a warmed (37 ºC) petri dish containing HBSS buffer and small excisions were made to release the sperm cells. After 5 min, to allow sperm cells to flow out of the epididymis to the HBSS buffer, 1 mL of the HBSS + sperm solution was collected for sperm parameter analysis and sperm RNA extraction. The amount of sperm debris and other cells observed in these sperm swabs was negligible. A) F_0-CTRL; B) F_0-HFD; C) F_0-HFD$_t$; D) F_1-CTRL; E) F_1-HFD; F) F_1-HFD$_t$; G) F_2-CTRL; H) F_2-HFD; I) F_2-HFD$_t$.

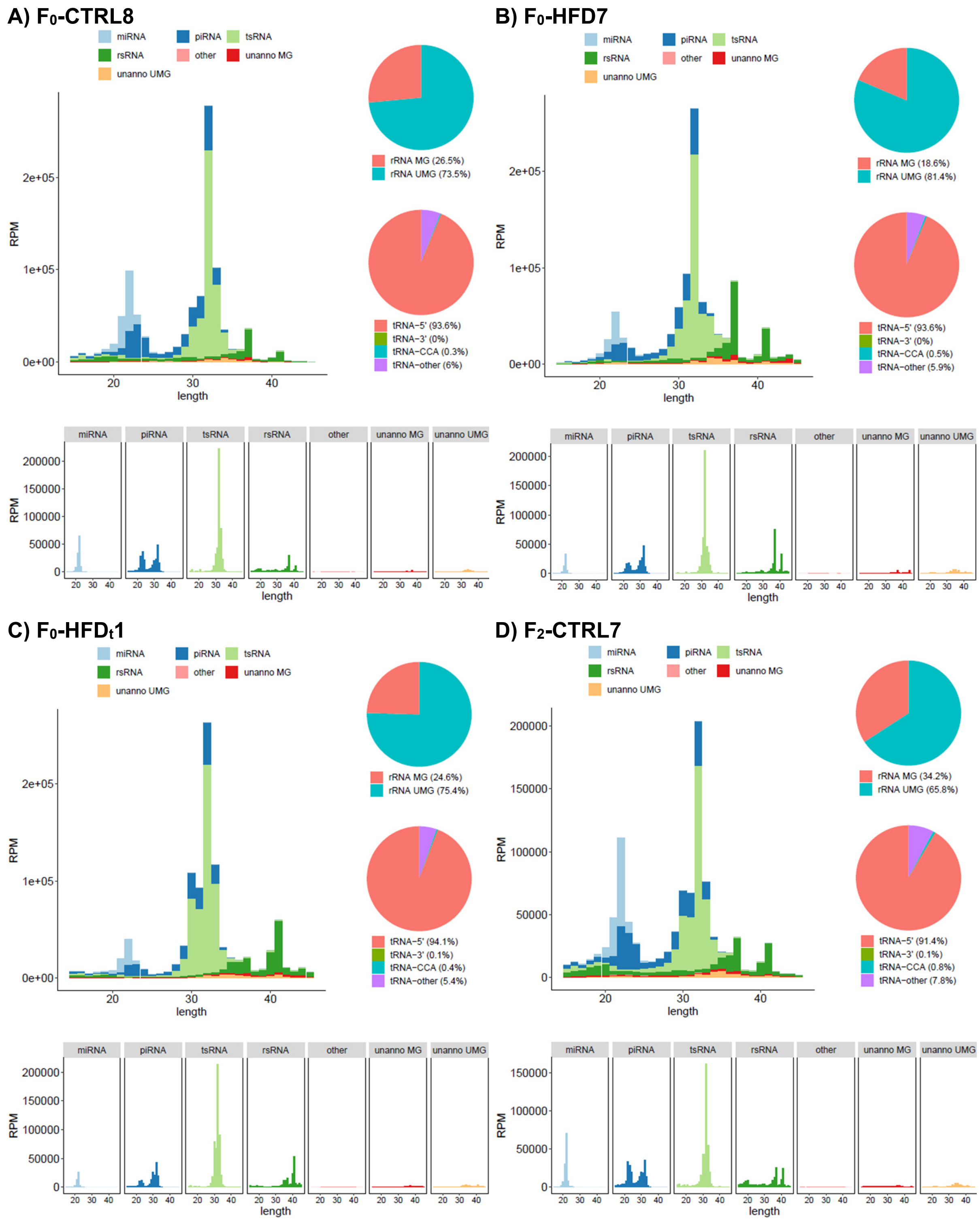

Figure S2: Representative sperm RNA diagnosis statistics obtained by SPORTS 1.1. The most abundant sncRNA biotype in sperm RNA extracts was tsRNA, similarly as reported by similar studies. miRNA, piRNA and rsRNA were present in similar proportions across samples. As expected, there are two distinct piRNAs populations, one 20-23 nucleotide long and another 28-32 nucleotide long. rsRNAs have a similar distribution at 35-38 nucleotides and 41-43 nucleotides. The proportion of rRNA matched to genome ranges from 20-35%, eliciting a low degree of contamination by somatic cells. A) F_0-CTRL8; B) F_0-HFD7; C) F_0-HFD_t1; D) F_2-CTRL7.

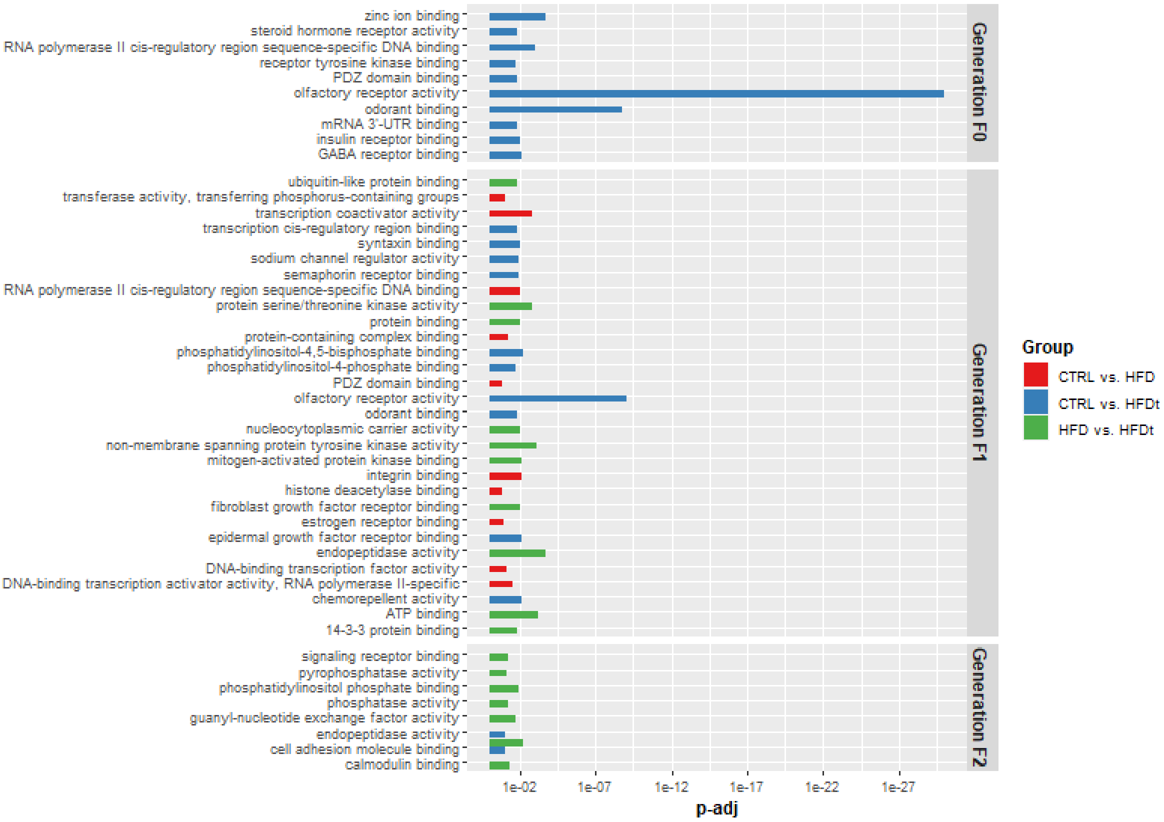

Figure S3: Gene ontology of the targets of differently expressed sperm sncRNAs, according to Molecular Function. Targets of sncRNAs were estimated recurring to the online suite sRNAtools (https://bioinformatics.caf.ac.cn/sRNAtools/). The output was then used for functional annotation based on the Gene Ontology Resource (http://geneontology.org/). The annotation was performed by the topGO package ran in R 4.1.0. GO terms with less than 10 annotated genes and single gene targets were excluded from the analysis. CTRL - mice fed with standard diet and their offspring; HFD – mice fed with high-fat diet and their offspring; HFDt – mice fed with high-fat diet for 60 days then replaced by standard diet and their offspring.

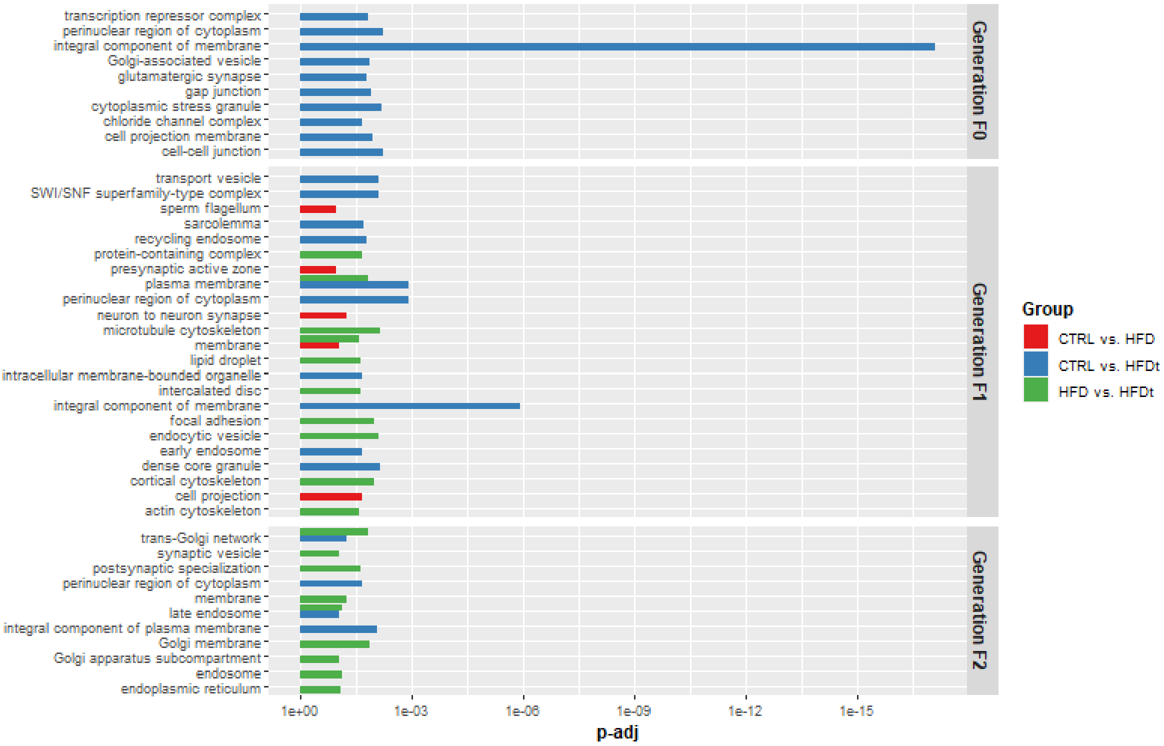

Figure S4: Gene ontology of the targets of differently expressed sperm sncRNAs, according to Cellular Component. Targets of sncRNAs were estimated recurring to the online suite sRNAtools (https://bioinformatics.caf.ac.cn/sRNAtools/). The output was then used for functional annotation based on the Gene Ontology Resource (http://geneontology.org/). The annotation was performed by the topGO package ran in R 4.1.0. GO terms with less than 10 annotated genes and single gene targets were excluded from the analysis. CTRL - mice fed with standard diet and their offspring; HFD – mice fed with high-fat diet and their offspring; HFDt – mice fed with high-fat diet for 60 days then replaced by standard diet and their offspring.

Table S1: Detailed list of differently expressed sperm sncRNAs.

miRNA	tsRNA		piRNA	repRNA
	tRF	tiRNA		
3068-5p, 215-5p, 144-3p, 196a-5p, 196b-5p, 34c-5p, 471-5p, 127-3p, 135b-5p, 32-5p, 145a-5p	mt_Gln-TTG_5_end, mt_Lys-TTT_5_end, mt_Asn-GTT, iMet-CAT, mt_Trp-TCA_CCA_end, Ala-TGC_5_end, Gln-CTG_5_end, Gln-CTG_CCA_end, iMet-CAT_5_end, Leu-CAA_5_end, Leu-TAA_5_end, Val-AAC_5_end, Val-CAC_5_end, Pro-AGG_3_end, Pro-CGG_3_end, Pro-TGG_3_end	Val-AAC, mt_Gly-TCC, mt_Gly-TCC_CCA_end, mt_His-GTG_3_end, mt_His-GTG_CCA_end, mt_Lys-TTT_CCA_end, mt_Met-CAT_5_end, mt_Pro-TGG_CCA_end, mt_Ser-GCT_CCA_end, mt_Val-TAC_CCA_end	Acvr2b, Ankrd11, BC026590, Bcl2l13, Cbfa2t2, Cramp1l, Ing5, Ipmk, Klhl11, Ppp1r12b, Rab11fip4, Nsd1, Shank3, Strbp, Tef, Tfcp2l1, Uhrf1bp1, Wipf2, 4-qD2-349	L1Md_T, L1Md_F2, ID4_, (TATATG)n, 7SLRNA, U1, FordPrefect, U5, U4, RLTR10, L1MA5, L2c, MER5B, MLT2B4, (CAG)n, MTE2a-int, GC_rich, MLT1A1, RMER12, RLTR11B, MTD-int, (TCCA)n, MER33, RLTR24, Tigger15a, MARNA, BGLII, L1MA9, Charlie5, RMER6A, MLT2B3, RMER17C-int, MLT1J2, Tigger17c, Tigger13a, L1MEc, L1M4a2, MER113, MLT1F2, (ATTG)n, RMER6D, MuRRS4-int, LTR41B, MER115, MER58C, RMER17A2, RLTR14-int, RMER20A, Arthur1, RMER6B, MLT2F, IAPEY4_I-int, L1VL2, RLTR13G, AmnSINE1, MLT1H2, RLTR30B_MM, RLTR26D_MM, L1MCa, L1MB1, L1ME3, MMETn-int, L4_B_Mam, RLTR12E, MTEb-int, RLTR13D1, Charlie24, IAPEY2_LTR, ORR1A1-int, MamTip2, MER102b, IAP-d-int, ORR1C2-int, RLTR18, RLTR12D, MER94B, MamRep1894, MamGypLTR1d, RLTR13D6, MER135, L1MCb, ERVB2_1A-I_MM-int, MER68-int, LTRIS4B, BGLII_C, MER91C, LTRIS4, RLTR18-int, LTR86C, LTR28, IAPLTR3-int, (CATA)n, RLTR20A4, HY1, ERV3-16A3_I-int, MER90a, Ricksha_c, LTR10_RN, RLTR13D2, ORR1F-int, RLTR9A

Filename: biomedicines-1651197-supplementary.docx
Directory: E:\4.15\polymers-1660052
Template: C:\Users\MDPI\AppData\Roaming\Microsoft\Templates\Normal.dotm
Title:
Subject:
Author: Luís Crisóstomo
Keywords:
Comments:
Creation Date: 11/1/2021 8:59:00 PM
Change Number: 40
Last Saved On: 4/14/2022 11:58:00 AM
Last Saved By: MDPI
Total Editing Time: 681 Minutes
Last Printed On: 4/15/2022 4:24:00 PM
As of Last Complete Printing
Number of Pages: 6
Number of Words: 750 (approx.)
Number of Characters: 4,133 (approx.)
